# Nurses' Perceptions and Practices Toward Clinical Alarms in a Transplant Cardiac Intensive Care Unit: Exploring Key Issues Leading to Alarm Fatigue

**DOI:** 10.2196/humanfactors.4196

**Published:** 2015-03-16

**Authors:** Azizeh Khaled Sowan, Albert Fajardo Tarriela, Tiffany Michelle Gomez, Charles Calhoun Reed, Kami Marie Rapp

**Affiliations:** ^1^ University of Texas Health Science Center at San Antonio School of Nursing, Department of Health Restoration and Care Systems Management San Antonio, TX United States; ^2^ University Health System Transplant Cardiac ICU San Antonio, TX United States; ^3^ University Health System Center for Nursing Excellence San Antonio, TX United States; ^4^ University Health System Nursing Administration San Antonio, TX United States

**Keywords:** clinical alarms, alarm fatigue, critical care, physiologic monitors, nursing, survey

## Abstract

**Background:**

Intensive care units (ICUs) are complex work environments where false alarms occur more frequently than on non-critical care units. The Joint Commission National Patient Safety Goal .06.01.01 targeted improving the safety of clinical alarm systems and required health care facilities to establish alarm systems safety as a hospital priority by July 2014. An important initial step toward this requirement is identifying ICU nurses’ perceptions and common clinical practices toward clinical alarms, where little information is available.

**Objective:**

Our aim was to determine perceptions and practices of transplant/cardiac ICU (TCICU) nurses toward clinical alarms and benchmark the results against the 2011 Healthcare Technology Foundation’s (HTF) Clinical Alarms Committee Survey.

**Methods:**

A quality improvement project was conducted on a 20-bed TCICU with 39 full- and part-time nurses. Nurses were surveyed about their perceptions and attitudes toward and practices on clinical alarms using an adapted HTF clinical alarms survey. Results were compared to the 2011 HTF data. Correlations among variables were examined.

**Results:**

All TCICU nurses provided usable responses (N=39, 100%). Almost all nurses (95%-98%) believed that false alarms are frequent, disrupt care, and reduce trust in alarm systems, causing nurses to inappropriately disable them. Unlike the 2011 HTF clinical alarms survey results, a significantly higher percentage of our TCICU nurses believed that existing devices are complex, questioned the ability and adequacy of the new monitoring systems to solve alarm management issues, pointed to the lack of prompt response to alarms, and indicated the lack of clinical policy on alarm management (*P*<.01). Major themes in the narrative data focused on nurses’ frustration related to the excessive number of alarms and poor usability of the cardiac monitors. A lack of standardized approaches exists in changing patients’ electrodes and individualizing parameters. Around 60% of nurses indicated they received insufficient training on bedside and central cardiac monitors. A correlation also showed the need for training on cardiac monitors, specifically for older nurses (*P*=.01).

**Conclusions:**

False and non-actionable alarms continue to desensitize TCICU nurses, perhaps resulting in missing fatal alarms. Nurses’ attitudes and practices related to clinical alarms are key elements for designing contextually sensitive quality initiatives to fight alarm fatigue. Alarm management in ICUs is a multidimensional complex process involving usability of monitoring devices, and unit, clinicians, training, and policy-related factors. This indicates the need for a multi-method approach to decrease alarm fatigue and improve alarm systems safety.

## Introduction

Clinical alarms are the top hazard listed in the 2014 Emergency Care Research Institute’s (ECRI) “Top Ten Health Technology Hazards” report [1]. Consensus exists on research about the low specificity and excessive number of false alarms (86%-99.5%) produced by physiological monitors [2-4]. This results in clinicians ignoring or disabling alarms, a phenomenon known as alarm fatigue, and raises a question about the clinical value of the currently used physiological alarm systems.

Fatal incidents related to clinical alarms are well documented [5,6]. As a result, the Joint Commission (JC) National Patient Safety Goal (NPSG .06.01.01) targeted improving the safety of clinical alarm systems, requiring health care facilities to establish alarm systems safety as a hospital priority by July 2014 [7].

A 2005-2006 national survey of more than 1300 health care professionals and other hospital personnel (such as monitor technicians and clinical engineers) by the Healthcare Technology Foundation (HTF) Clinical Alarms Committee showed that nuisance alarms are frequent (81%), disrupt care (77%), and reduce trust in alarms, causing clinicians to inappropriately disable them (78%) [8]. Newer-monitoring systems did not solve alarms problems (69%) [8]. The HTF clinical alarms survey is the most comprehensive survey available to date, in comparison to other surveys measuring perceptions and attitudes toward clinical alarms [9,10]. The HTF clinical alarms survey was developed by a group of multidisciplinary experts in biomedical engineers, safety, and instrumentation and was supported for administration by different safety and regulatory agencies such as the Association for the Advancement of Medical Instrumentation, Food and Drug Administration/MEDSUN, ECRI, and others [8]. Surprisingly, a 2011 administration of the same survey to a larger group of clinicians (N=4278) revealed very similar results [11]. It is worth noting that the combined samples of 2005-2006 and 2011 HTF clinical alarms surveys were from more than ten different hospital departments, including the intensive care units (ICUs).

Monitoring the physiological condition of critically ill patients is a complex task where the use of multiple monitoring devices per patient is the norm and an essential component in the treatment process. Nurses are the key professionals responding to alarms and managing the multiple monitoring devices [5]. The high rate of false alarms constantly reported in ICUs [12-14] compared to non-critical care units [15] resulted in nurses responding to an average of 150-400 alarms per patient per day in ICUs [16]. Therefore, alarm safety is a clear priority in these units. This also suggests that ICU nurses may have different perceptions toward clinical alarms than nurses in other clinical areas. Thus, nurses’ attitudes toward clinical alarms and their perceptions of factors that may threaten alarm recognition and response are essential in guiding research projects and quality initiatives for alarm management in ICUs.

None of the available studies on perceptions and attitudes to alarm management have yet to benchmark their results with the HTF clinical alarms national data using the complete version of the survey. Little information is available about ICU nurses’ attitudes and common practices related to clinical alarms [9,10,17], and none is available specifically about nurses’ perceptions and attitudes in transplant/cardiac ICU (TCICU), the target setting of this project. Examining nurses’ attitudes and practices toward clinical alarms using a comprehensive survey such as the HTF clinical alarms survey is essential to understand the complexity of the ICU work environment and contributing factors that threaten the safety of alarm recognition and appropriate management. Additionally, in the project setting, state-of-the-art new physiological monitoring devices are used, demanding an evaluation of their capabilities to reduce alarm fatigue and improve alarm safety.

Nurses on a 20-bed TCICU identified an excessive number of clinical alarms, specifically from the cardiac monitors, as a safety hazard that caused work disruption. An interprofessional alarm management taskforce consisting of nurses, physicians, and biomedical engineers was assembled to attain phase 1 A. of the JC NPSG.06.01.01, to establish alarm systems safety as a hospital priority. The initial project goal was to standardize alarm management in all ICUs. This phase of the project had two objectives: (1) to determine TCICU nurses’ perceptions and attitudes toward clinical alarms signal from all physiological monitors, as well as current practices and educational needs for alarm management using the cardiac monitors, and (2) to benchmark the results with the 2011 HTF clinical alarms survey data. Correlations between attitudes, nurse characteristics, and other factors such as training on monitoring devices were also examined for further insight into the current problem.

## Methods

### Design, Sample, and Setting

Approval to conduct this quality improvement project was obtained from the hospital’s Institutional Review Board, and implied consent was obtained from the participating nurses. This project was conducted on a 20-bed TCICU located in a 684-bed university teaching Magnet hospital in the Southwestern United States. The unit has 39 full- and part-time nurses with a nurse patient ratio of 1:2. The unit is equipped with modern patient monitoring devices (eg, cardiac monitors, pulmonary artery catheter monitoring, pulse oximeter) and with intensive care equipment for life support (eg, ventilator, infusion pump). The ICU is an “E” shape with patients’ rooms to the sides and an unstaffed central monitor station. At the time of this project, the unit had no policy for clinical alarm management.

In April 2014, the unit witnessed two major changes: the implementation of new cardiac monitors (Philips IntelliVue MX800) and Wi-Fi (CISCO) phones for communication. When deploying any physiological monitoring devices, nurses usually receive a group-based presentation with hands-on training on the device’s appropriate use by the device company representative. Device manuals are also available in the unit for nurses to review. Newly hired nurses are trained on device use during their orientation program by their preceptors, unit educators, or the company representatives. Usually, no other structured periodic training on managing physiological monitoring devices is offered. However, the nursing unit educators do provide individualized help for device management if needed. This project began 2.5 months after implementing the new cardiac monitors.

### Instrument and Procedure

We adapted the 2011 HTF clinical alarms survey after obtaining approval from the developers for its use to understand TCICU nurses’ attitudes and practices related to clinical alarms. Four expert ICU nurses reviewed the survey after adaptation for face validity and appropriateness to use in the TCICU. The adapted survey included three sections: (1) demographics, (2) perception about clinical alarms signaled from all monitoring devices, and (3) potential issues that interfere with alarm recognition. Section 2 in the HTF clinical alarms survey had 20 statements rated using a 5-point Likert-type scale of agreement followed by a free-text area to provide details on statements. Our changes to the HTF clinical alarms survey items involved Section 2 and consisted of (1) deleting the statement “The integration of clinical alarms into the Joint Commission patient safety measures have reduced patient adverse events” because it is not applicable to our setting yet, (2) adding three statements to capture other alarm issues specific to the TCICU related to the types of alarms, alarm specificity, and the unit layout, and (3) replacing “institution” and “floor/area of the hospital” to “unit” in some statements, to reflect the context of measurement. Section 3 has 9 issues to order in rank from 1 (most important) to 9 (least important).

Since the HTF clinical alarms survey is designed to measure clinicians’ attitudes toward alarms signaled from all physiological monitoring devices and because the unit deployed new cardiac monitors, three additional questions were also asked to understand nurses’ practices toward clinical alarms specific to the cardiac monitors. These were related to the (1) frequency of individualizing alarms’ parameters, (2) frequency of changing electrodes, and (3) adequacy of the training received on using the cardiac monitors.

The survey was designed using SurveyMonkey and placed on a hospital website. In coordination with the nursing director of the TCICU, we sent individual recruitment emails, each with a unique ID, to all 39 TCICU nurses with a link to the adapted survey. ID numbers were used for follow-up on responses. Two email reminders were sent to non-respondents by the first author (AS), who is not employed at the project setting, to enhance the response rate.

### Data Analysis

Descriptive statistics were used to describe respondents’ characteristics (age, clinical, and computer experience) and to summarize questionnaire responses. A *Z* test for difference in proportions for independent samples was used to examine the difference between the percentages of HTF clinical alarms survey respondents and TCICU nurses in this project. Mean ranks were used for the ranking section of the survey. Content analysis was used to categorize the narrative data into themes. Bivariate correlations between demographic information and other survey statements and questions related to training and practices were calculated using a chi-square test. A level of significance of .05 was used for all statistical tests.

## Results

### Overview

A total of 39 completed responses (100% response rate) were obtained with usable data. The majority of nurses were females (25/39, 64%), about 40 years old (28/39, 72%), and full-time staff (33/39, 85%). The percentages of nurses who reported having “1-3” and “>5” years of overall nursing experience were equal (16/39, 41%, in each category). However, the majority of nurses were within 1-3 years of TCICU experience (28/39, 72%). The mean score of the reported computer skills was 2.4 (SD 0.68) out of a 4-point Likert-type scale.

### General Statements About Clinical Alarms

Table 1 presents the percentages of TCICU nurses and HTF clinical alarms study respondents who agreed/strongly agreed on each of the 22-item statements about clinical alarms. A major assumption of *Z* test is that “n*p and n(1-p) must both be equal to or greater than 5”, where n is the sample size and p is the proportion. This assumption was not met when a very high percentage of our participants agreed/strongly agreed with several item statements; therefore, *Z* test for the difference in proportions was not calculated for the first five items.

Similar to the majority of the HTF study sample, almost all of our TCICU nurses agreed or strongly agreed on the first five statements regarding the frequency of nuisance alarms and the need for distinctive alarm sounds and visual displays (Table 1). The majority of the respondents from the two studies were also supportive of the use of smart alarms, hiring dedicated central monitor alarm management staff, and integrating the alarms into wireless devices (Items 6, 7, 10, 11). Almost two thirds of our TCICU nurses indicated that the unit layout interferes with alarm recognition and management (Item 8), and only half agreed that lethal alarms are responded to promptly (Item 15).

In contrast to the HTF study results, a significantly higher percentage of our nurses pointed to confusion in locating an alarming device (Item 9), believed that existing devices are complex for setting alarms parameters (Item 12), questioned (disagreed with) the ability and adequacy of the monitoring systems to alert staff of changes in a patient’s condition (Item 14), doubted the sensitivity of the clinical staff to alarms (Item 17), and did not think that the monitoring devices provided distinct outputs (Item 19). Additionally, the majority of our nurses indicated a lack of requirements to document the individualization of patient parameters (Item 20) and the absence of clinical policies on alarm management (Item 21). Almost all nurses believed that the new monitoring systems have not solved most of the previous problems they experienced with clinical alarms (Item 22).

**Table 1:** Percentages of TCICU nurses who agreed or strongly agreed on clinical alarm survey statements compared with respondents of the 2011 HTF survey data.

#	Statement	TCICU n^a^ (%)	HTF 2011 n^b^ (%)	*P*
1	Nuisance alarms disrupt patient care	38 (98)	4125 (71)	NA^c^
2	Nuisance alarms reduce trust in alarms and cause caregivers to inappropriately turn alarms off at times other than setup or procedural events	38 (98)	4133 (78)	NA^c^
3	Alarm sounds and/or visual displays of the current monitoring systems and devices should clearly differentiate the priority of alarm	37 (95)	4137 (91)	NA^c^
4	Alarm sounds and/or visual displays should be distinct based on the parameter or source (eg, device)	37 (95)	4130 (91)	NA^c^
5	Nuisance alarms occur frequently	37 (95)	4124 (77)	NA^c^
6	Smart alarms (eg, where multiple parameters, rate of change of parameters, and signal quality are automatically assessed in their entirety) would be effective to use for improving clinical response to important patient alarms	31 (80)	3783 (78)	.7
7	Smart alarms (eg, where multiple parameters, rate of change of parameters, and signal quality are automatically assessed in their entirety) would be effective to use for reducing false alarms	30 (78)	3791 (78)	.9
8^d^	Unit layout does interfere with alarm recognition and management	28 (73)	NA	NA
9	When a number of devices are used with a patient, it can be confusing to determine which device is in an alarm condition	28 (73)	3916 (51)	<.01^e^
10	Central alarm management staff responsible for receiving alarm messages and alerting appropriate staff is helpful	24 (59)	3890 (53)	.4
11	Alarm integration and communication systems via pagers, cell phones, and other wireless devices are useful for improving alarms management and response	23 (56)	3786 (56)	.9
12	Properly setting alarm parameters and alerts is overly complex in existing devices	22 (56)	4009 (21)	<.001^e^
13	Environmental background noise has interfered with alarm recognition	21 (54)	3919 (42)	.1
14^f^	The alarms used on my unit are adequate to alert staff of potential or actual changes in a patient’s condition	20 (51)	3978 (72)	<.001^e^
15^d^	When a lethal alarm sounds, it is clearly and quickly recognized and immediate action is taken to address the alarm	19 (49)	NA	NA
16^d^	Nearly all alarms are actionable (requiring the nurse to respond and take an action)	19 (49)	NA	NA
17	Clinical staff is sensitive to alarms and responds quickly	13 (34)	3935 (66)	<.001^e^
18	There have been frequent instances where alarms could not be heard and were missed	12 (32)	3999 (29)	.6
19^f^	The medical devices used on my unit all have distinct outputs (ie, sounds, repetition rates, visual displays) that allow users to identify the source of the alarm	12 (32)	3927 (70)	<.001^e^
20^f^	There is a requirement in my unit to document that the alarms are set and are appropriate for each patient	12 (29)	3784 (71)	<.001^e^
21^f^	Clinical policies and procedures regarding alarm management are effectively used in my unit	8 (20)	3772 (55)	<.001^e^
22	Newer monitoring systems (eg, <3 years old) have solved most of the previous problems we experienced with clinical alarms	1 (2)	3988 (29)	<.001^e^

^a^This “n” reflects only the participants who agreed/strongly agreed on each statement and not the total sample size. The total sample size was 39.

^b^This “n” is the number of respondents who answered each statement and is not limited to those who agreed/strongly agreed on each statement, and was used to calculate *Z* test. These numbers are unpublished data and were obtained from the HTF. The total sample size of the 2011 HTF survey is 4278.

^c^NA= Not applicable. No *Z* scores were calculated for difference between the two studies on these statements because “n*p and n(1-p)” were less than 5.

^d^These are the new statements that we added to our survey and were not available in the HTF survey. Therefore, no *Z* score was calculated.

^e^Significant at *P*<.05.

^f^These are the statements where the “floor/area of the hospital” or “institution” in the HTF clinical alarms survey were replaced with “unit”.

### Narrative Data

A total of 22 nurses provided narrative comments about clinical alarms and issues threatening timely recognition and response. Categories, themes, and examples of comments are listed in Table 2. All comments were negative reflecting serious issues related to safety; poor usability of the cardiac monitors; a lack of support to the use of evidence-based solutions for alarm management, such as watchers for the central monitors and connecting alarms of the monitoring devices to the communication devices (eg, CISCO phones) [5,18]; and unit-related factors, such as a lack of policy to manage alarms, unit layout interferes with alarm response, and the need for further training on the cardiac monitors.

**Table 2:** Categories, themes, and comments of the TCICU nurses’ narrative data (N=22).

Categories and themes		Examples of comments
**Category 1: Frequent false alarms and patient safety**		
	Theme 1: False alarms are very frequent and very distracting (12 nurses)	“too much alarms that distract care and patient sleep”
		“they signal for no reason even in an empty patient room”
		“the continuous "bing" of the central monitor gives me a huge headache”
	Theme 2: There is a tendency by nurses to ignore clinical alarms (5 nurses)	“the nuisance of the new cardiac monitors is so overwhelming you tend to ignore”
		“I have watched multiple nurses at the nursing desk listen to alarms sounding and not respond, very worrisome”
**Category 2: Poor usability of the medical devices**		
	Theme 3: Alarms’ sounds and visual displays are not distinct based on the priority of the alarm, parameter, or the device (9 nurses)	“lethal alarms are not distinguishable than other alarms”
		“alarms’ sounds and visual displays sound and look alike for different vitals”
	Theme 4: The new cardiac monitors are very complex and not user friendly (4 nurses)	“newer cardiac monitors made it worst, they are just fancier”
		“cardiac monitors are too difficult to navigate, and takes away time to care for patient which is more important than figuring the monitor to function, they are FOREVER alarming”
		“I am unable to correct false alarms easily”
		“alarms will sound for false Vtachs with no way to silence or relearn”
		“cardiac monitor can't recognize the waveform of SPO2, adjustment on wave height is necessary”
	Theme 5: The lowest volume of the alarms is still very loud and distracts patient sleep (2 nurses)	“alarms are very loud within the room, even turning the volume down to the lowest level is still loud- keeps patients awake at night”
		“the new cardiac monitors have the same volume alarm for even the most trivial alarms that it sets a cry wolf mentality and could pose a dangerous situation in which an actual true alarm could be disregarded”
**Category 3: Lack of support to the use of evidence-based solutions for alarm management**		
	Theme 6: A central monitor watcher will not solve the problem (3 nurses)	“having a watcher might be unsafe, will relax the monitoring eyes/ears of a nurse as a another person is equally monitoring”
		“it will add to alarm fatigue, it would be easier for me to just go in the room and fix the problem than have someone constantly calling me”
	Theme 7: Unreliable technology to integrate with alarms (3 nurses)	“CISCO phones and pagers sometimes don’t alert or receive any alarms even for emergencies, there are delays on them and they loose the signals in the elevators”
**Category 4: Unit-related factors to alarm management**		
	Theme 8: Absence of alarm management and documentation policy (3 nurses)	“we need to reinforce that alarm parameters need to be changed specific to the patient”
		“there is no place in the medical record to document that alarms are individualized based on patient condition”
	Theme 9: Unit layout may hinder response to alarms (2 nurses)	“although alarms are loud within the patient room, the E-shape unit makes the unit too large and resulted in alarms being unheard”
		“even within the same hallway a fatal alarm can be missed”
		“with the big unit, we cannot see all patients in the central monitor unless adjustment is done”
	Theme 10: Further training on monitoring devices is required (1 nurse)	“there is not enough time to train staff on the central monitor alarm”

### Ranking of Issues that Affect Alarm Recognition

Table 3 presents ranking of the issues that may affect alarm recognition and response by TCICU nurses and HTF study respondents. The top four critical issues identified by TCICU nurses endangering alarm recognition and response were similar to the HTF data. However, the rankings of these issues differed according to our nurses who, for example, ranked “difficulty in identifying the source of an alarm” as the first critical issue versus the HTF respondents who ranked this issue as second. Interestingly, and similar to the HTF study, our nurses ranked the lack of training as one of the three least important issues, as well as noise competition from nonclinical alarms.

**Table 3:** Ranking of TCICU nurses compared to respondents of the 2011 HTF clinical alarms survey on the importance of issues that affect response to alarms (1=most important, 9=least important).

Items	Our ICU data (N=39)		HTF 2011 data (N=4276)	
	Mean^a^	Ranking^b^	Mean^a^	Ranking^b^
Difficulty in identifying the source of an alarm	2.94	1	4.61	2
Difficulty in understanding the priority of an alarm	3.06	2	4.64	3
Difficulty in hearing alarms when they occur	3.93	3	4.70	4
Frequent false alarms, which lead to reduced attention or response to alarms when they occur	4.15	4	4.21	1
Inadequate staff to respond to alarms as they occur	4.23	5	4.87	6
Difficulty in setting alarms properly	4.44	6	5.16	7
Noise competition from nonclinical alarms and pages	4.45	7	5.66	9
Over-reliance on alarms to call attention to patient problems	4.77	8	4.86	5
Lack of training on alarm systems	6.60	9	5.55	8

^a^Mean rank of the item.

^b^Ranking of the mean.

### Nurses’ Practices and Level of Training Related to Cardiac Monitors

**Figure 1:**
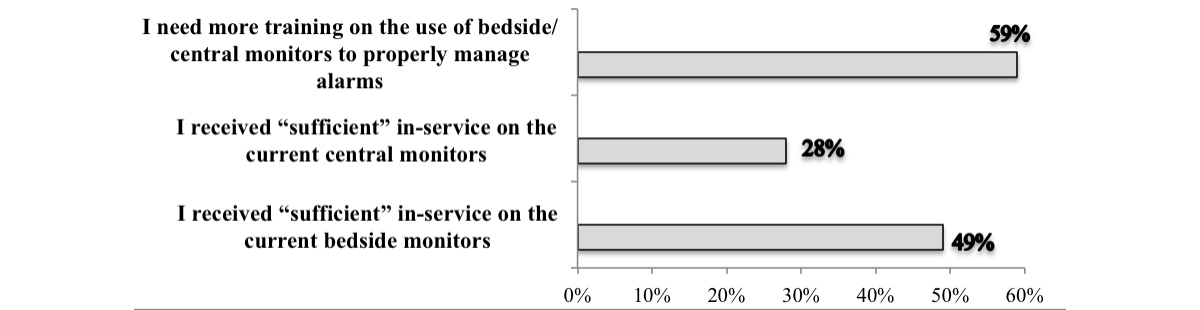
Percentages of TCICU nurses who agreed/strongly agreed on the adequacy of the training received on bedside and central cardiac monitors (N=39).

The results support the lack of standardized approaches in changing patients’ electrodes and individualizing parameters. Only half of the nurses reported changing electrodes every 24 hours (51%, 20/39 nurses). Other nurses reported changing electrodes only when needed (23%, 9/39), every shift (13%, 5/39), or every 48 hours (13%, 5/39). Similarly, more than one third of the nurses indicated not changing monitors’ parameters, and only 5% (2/39) change parameters after disconnecting the patient from the monitor and when the setting reverted to defaults. Over half of the nurses indicated the need for more training on the bedside and central cardiac monitors (Figure 1).

### Correlations

Bivariate correlations using chi-square test were examined between age, computer skills, years of ICU experience (as the demographic data), and the perception about the complexity of the monitoring devices (survey Item 12), adequacy of alarms to alert staff (Item 14), frequency of changing patient parameters and electrodes, and the need for further training on cardiac monitors. All variables were recoded as binary. None of the correlations were significant, except for the positive relationship between age and the need for further training on cardiac monitors (*P=*.01).

## Discussion

### Overview

A mountain of evidence exists on the need for alarm management [19]. However, the majority of the available studies targeted changing specific parameters or new algorithms and their effect on decreasing the number of false alarms and were not guided by issues recognized by clinicians as critical for alarm management and response [12-14,20-23]. Identifying key issues leading to alarm desensitization as a safety threat should be a priority in alarm management. This project focused on understanding the context-related attitudes and practices of TCICU nurses toward clinical alarms and benchmarked the results with the 2011 HTF clinical alarms national data. The results showed that alarm fatigue is a critical and urgent issue in our TCICU. In comparison to the HTF study results, the responses of our TCICU nurses highlighted the complexity of alarm management in ICUs.

### Principal Findings

This project helped identify key issues leading to alarm desensitization. Our nurses and the HTF study respondents agree that false alarms occur frequently, disrupt care, and reduce trust in alarm systems and response sensitivity, causing clinicians to inappropriately disable them. On the other hand, and unlike the HTF data, the majority of our TCICU nurses challenged the ability and adequacy of the new monitoring systems in solving these alarm management issues. Narrative data attributed this primarily to usability issues with the devices, specifically the cardiac monitors. These were non-trivial issues such as the complexity in navigation to set alarm parameters, the inability of the nurse to turn off some of the false alarms or to adjust alarm volume, failure of the monitors to identify that patients were disconnected from monitors and were alarming in empty rooms, failure of the monitors to display the waveforms of the parameters in their appropriate size, and the look-alike and sound-alike alarms for different parameters with different priorities and from different devices. Most important, these issues affected the timely recognition of lethal alarms, resulting in only 50% of the nurses reporting prompt response to such alarms.

The comments related to poor usability and lack of user-centered devices were all linked to the new cardiac monitors, indicating the need for future research on usability testing even for the newest devices and especially for complex ICU monitoring devices that may jeopardize safety and workflow efficiency. Little information is available about the usability of physiological monitoring devices [24,25]. Unlike previous studies on alarm fatigue [9,10], usability of the monitoring devices was a major reason behind nurses’ frustration with alarm systems in this project. These findings are congruent with the fast pace, high-stress level, and complexity of the ICUs, where monitoring devices need to be useful tools to guide clinical decision-making rather than positive contributors to the stress level, workload, workflow inefficiency, and sleep deprivation among patients.

One of the nurses described the cardiac monitor as a “fancy” monitor, suggesting the availability of unused features by clinicians and perhaps the lack of knowledge on the appropriate use and usefulness of some features. For patient safety in ICUs, previous research supported the need to eliminate unnecessary alarms [22] and to understand triggered defaults. In fact, overuse of alarms of the monitoring devices is a practice associated with overdiagnosis; it may do more harm than good. Finding only 5% of our nurses changing alarm parameters after disconnecting the patient from the monitor may indicate that nurses are unaware that the disconnection results in settings reverting to defaults (a critical safety feature).

Results showed that our nurses were supportive of employing a dedicated person for the central monitor and the integration of alarms into the communication devices [5]. However, narrative comments highlighted safety and feasibility concerns. This indicates the need to pilot any initiatives for alarm management to assure their appropriateness.

Another unique finding of this project was that unit layout was a major factor interfering with alarm response and recognition. Instead, unit architectural layout should be a facilitator to timely response and recognition. Nurse input to unit design is imperative in the future. Additionally, alarm policies and requirements to document alarm settings were absent. An alarm management policy could eliminate non-standardized practices related to frequency of changing the electrodes and customizing patients’ parameters. The American Association of Critical Care Nurses’ evidence-based recommendation of electrode change is “daily and if needed” [26]. Only 50% of our nurses were following this recommendation. Also, more than a third of our nurses reported not customizing alarm parameters to be patient specific. These practices contribute significantly to increasing the number of false alarms in ICUs [5], but examining whether nurses have sufficient knowledge on parameter limits is equally important. While 44% of the TCICU nurses customize the parameters, evidence-based hard stops are needed in these devices, specifically for critical parameters.

The rankings assigned to the importance of issues identified the source of an alarm and understanding its priority as the top two critical issues, reflecting the complexity of the monitoring devices and their current inadequacy. Difficulty in hearing alarms, ranked as the third most important issue, may be attributed to the unit layout as explained by nurses in the narrative comments. The lack of training was listed as the least important factor, but in contrast, 60% of the nurses doubted their abilities to manage cardiac monitors and requested further training. This may be because the training question was limited to the cardiac monitors while survey items concerned all existing devices. Most important, nurses’ responses reflect the high frustration level of nurses who think that devices should be designed to be easy to use at a minimum and should help nurses acknowledge the source and priority of the alarms without the nurse spending time figuring out basic operational issues. Furthermore, the need for further training may also reflect deficiencies in the current group-training method, suggesting techniques such as the use of simulation [5], periodic refresher training, and super users. Interestingly, our results also supported a positive correlation between age and the need for training. This indicates that training methods may need to be revised for older nurses or that older nurses might be more resistant to change.

### Summary and Future Directions

Our results highlight the complexity of overall alarm management in ICUs and that ICU nurses may have different perceptions toward alarm management than other nurses. Appropriate alarm management depends on a combination of device usability, training, unit layout, IT infrastructure, and alarm management protocols and documentation capabilities [23,27]. In summary, this complexity suggests that (1) policies should be in place to guide end users of monitoring devices on alarm management, (2) device usability is fundamental for alarm management and emphasis in this area is needed, (3) the traditional group-based, one-round training on complex alarm-equipped monitoring devices is inadequate, (4) a need exists for structured evaluation of quality initiatives to ensure their appropriateness for different work cultures, and (5) focusing on one strategy (eg, changing alarms’ algorithms) to decrease false alarms may be insufficient to improve alarm fatigue.

### Limitations

The findings of this project can be generalized with caution. We obtained a 100% response rate, indicating a motivated sample, perhaps reflecting the importance of this issue to ICU nurses, the high stress level experienced by our nurses toward clinical alarms, and the need for urgent initiative to manage this problem. However, the sample size is relatively small and the results are limited to a TCICU in one setting with monitors from specific vendors. Other frequently used devices in other ICUs may also contribute negatively or positively to alarm fatigue. Including other ICUs will also increase the sample size. We measured nurses’ attitudes 2.5 months after the introduction of the new cardiac monitors because we thought this time period would be sufficient for nurses to adapt to the new devices. Measuring attitudes before or after that time period might reveal other findings as a result of novelty in using the devices (if measured before) or adaptation to the new monitors (if measured after). Last, although we could not use the *Z* test to measure the difference between our nurses and the HTF respondents on the first five statements on the survey; the comparable high percentages of respondents from the two studies on these statements predict the absence of any statistical differences.

### Conclusions

Clinical alarm management is in its infancy in many institutes. False and non-actionable alarms continue to desensitize clinicians and may result in missed fatal alarms. A multi-method approach in decreasing alarm fatigue and improving alarm systems safety is needed across devices, training, unit layout, clinicians, and policies. Usability of monitoring devices is essential in alarm management. Clinicians’ attitudes and practices related to clinical alarms are key in designing contextually sensitive quality initiatives to fight alarm fatigue. Partnership between clinicians, organizations, researchers, manufacturers, safety, and regulatory organizations is essential to improve alarm management. In the future, a comparison across other ICUs is needed and comprehensive usability studies are essential.

## Abbreviations

ECRI: Emergency Care Research Institute
HTF: Healthcare Technology Foundation
ICU: intensive care unit
JC: Joint Commission
NPSG: National Patient Safety Goal
TCICU: transplant/cardiac ICU

## References

[1] ECRI.

[2] Imhoff Michael, Kuhls Silvia (2006). Alarm algorithms in critical care monitoring. Anesth Analg.

[3] Phillips Joanne, Barnsteiner Jane H (2005). Clinical alarms: improving efficiency and effectiveness. Crit Care Nurs Q.

[4] Atzema Clare, Schull Michael J, Borgundvaag Bjug, Slaughter Graham R D, Lee Cheong K (2006). ALARMED: adverse events in low-risk patients with chest pain receiving continuous electrocardiographic monitoring in the emergency department. A pilot study. Am J Emerg Med.

[5] Association for the Advancement of Medical Instrumentation.

[6] The Joint Commission (2013). Sentinel event alerts: Medical device alarm safety in hospitals.

[7] The JC (2014).

[8] Clinical Alarm Task Force, AACE Healthcare Technology Foundation (2007). Impact of clinical alarms on patient safety: A report from the American College of Clinical Engineering Healthcare Technology Foundation. J Clin Eng.

[9] Christensen Martin, Dodds Andrew, Sauer Josh, Watts Nigel (2014). Alarm setting for the critically ill patient: a descriptive pilot survey of nurses' perceptions of current practice in an Australian Regional Critical Care Unit. Intensive Crit Care Nurs.

[10] Graham Kelly Creighton, Cvach Maria (2010). Monitor alarm fatigue: standardizing use of physiological monitors and decreasing nuisance alarms. Am J Crit Care.

[11] Funk Marjorie, Clark J Tobey, Bauld Thomas J, Ott Jennifer C, Coss Paul (2014). Attitudes and practices related to clinical alarms. Am J Crit Care.

[12] Görges Matthias, Markewitz Boaz A, Westenskow Dwayne R (2009). Improving alarm performance in the medical intensive care unit using delays and clinical context. Anesth Analg.

[13] Siebig Sylvia, Kuhls Silvia, Imhoff Michael, Langgartner Julia, Reng Michael, Schölmerich Jürgen, Gather Ursula, Wrede Christian E (2010). Collection of annotated data in a clinical validation study for alarm algorithms in intensive care--a methodologic framework. J Crit Care.

[14] Siebig Sylvia, Kuhls Silvia, Imhoff Michael, Gather Ursula, Schölmerich Jürgen, Wrede Christian E (2010). Intensive care unit alarms--how many do we need?. Crit Care Med.

[15] Schmid Felix, Goepfert Matthias S, Kuhnt Daniela, Eichhorn Volker, Diedrichs Stefan, Reichenspurner Hermann, Goetz Alwin E, Reuter Daniel A (2011). The wolf is crying in the operating room: patient monitor and anesthesia workstation alarming patterns during cardiac surgery. Anesth Analg.

[16] HCPro (2011).

[17] Cvach Maria M, Frank Robert J, Doyle Pete, Stevens Zeina Khouri (2014). Use of pagers with an alarm escalation system to reduce cardiac monitor alarm signals. J Nurs Care Qual.

[18] ECRI (2014).

[19] Cvach Maria (2012). Monitor alarm fatigue: an integrative review. Biomed Instrum Technol.

[20] Burgess Lawrence P A, Herdman Tracy Heather, Berg Benjamin W, Feaster William W, Hebsur Shashidhar (2009). Alarm limit settings for early warning systems to identify at-risk patients. J Adv Nurs.

[21] Aboukhalil Anton, Nielsen Larry, Saeed Mohammed, Mark Roger G, Clifford Gari D (2008). Reducing false alarm rates for critical arrhythmias using the arterial blood pressure waveform. J Biomed Inform.

[22] Biot Loic, Holzapfel Laurent, Becq Guillaume, Mélot Christian, Baconnier Pierre (2003). Do we need a systematic activation of alarm soundings for blood pressure monitoring for the safety of ICU patients?. J Crit Care.

[23] Solet Jo M, Barach Paul R (2012). Managing alarm fatigue in cardiac care. Progress in Pediatric Cardiology.

[24] Drews FA (2008). Patient monitors in critical care: Lessons for improvement. Henriksen K, Battles JB, Keyes MA. editors. Advances in Patient Safety: New Directions and Alternative Approaches (Vol. 3: Performance and Tools).

[25] Hyman William A (2010). Human factors: should your medical devices require intensive care?. Crit Care Nurs Clin North Am.

[26] (2013). American Association of Critical Care.

[27] ECRI Institute (2007). The hazards of alarm overload. Keeping excessive physiologic monitoring alarms from impeding care. Health Devices.

